# Supplementary material for: The anxious bipolar phenotype: clinical complexity and treatment response
Source: Int J Bipolar Disord. 2026 Feb 26;14:12. doi: 10.1186/s40345-026-00415-z (PMC13038839; doi:10.1186/s40345-026-00415-z)
Supplement: Supplementary file 1 — Supplementary Material 1 [file 40345_2026_415_MOESM1_ESM.docx]

**Supplement**

**Supplementary Table 1**. Differences in pharmacotherapeutic prescriptions among individuals with bipolar disorder-I, with and without comorbid anxiety disorders (ANX).

| **Variable** | **Total**  **(N=1451)** | **NoANX**  **(N=573)** | **ANX**  **(N=878)** | **p-value*** |
| --- | --- | --- | --- | --- |
| *Current prescriptions* |  |  |  |  |
| Lithium | 1086 | 412 | 674 | 0.009 |
| n (%) | 459 (42.3%) | 209 (50.7%) | 250 (37.1%) |  |
| Lamotrigine | 1139 | 439 | 700 | 0.092 |
| n (%) | 382 (33.5%) | 134 (31.8%) | 248 (34.6%) |  |
| Valproic | 1124 | 436 | 688 | 0.137 |
| n (%) | 290 (25.8%) | 136 (31.2%) | 154 (22.4%) |  |
| Carbamazepine | 1057 | 397 | 660 | 0.830 |
| n (%) | 31 (2.9%) | 11 (2.8%) | 20 (3.0%) |  |
| Gabapentinoids | 1436 | 546 | 890 | 0.041 |
| n (%) | 95 (6.6%) | 27 (4.8%) | 68 (7.8%) |  |
| Benzodiazepines | 1327 | 505 | 822 | **<0.001** |
| n (%) | 464 (35.0%) | 140 (27.7%) | 324 (39.4%) |  |
| Non-BZD sedatives | 1316 | 502 | 814 | 0.566 |
| n (%) | 105 (8.0%) | 37 (7.4%) | 68 (8.4%) |  |
| SGA | 1405 | 548 | 857 | 0.607 |
| n (%) | 809 (57.6%) | 319 (58.2%) | 490 (57.2%) |  |
| Olanzapine | 1087 | 426 | 661 | 0.023 |
| n (%) | 134 (12.3%) | 72 (16.9%) | 62 (9.4%) |  |
| Clozapine | 1066 | 415 | 651 | 0.623 |
| n (%) | 14 (1.3%) | 5 (1.2%) | 9 (1.4%) |  |
| Quetiapine | 1131 | 434 | 697 | 0.266 |
| n (%) | 314 (27.8%) | 121 (27.9%) | 193 (27.7%) |  |
| Aripiprazole | 1101 | 424 | 677 | 0.922 |
| n (%) | 187 (17.0%) | 68 (16.0%) | 119 (17.6%) |  |
| Risperidone | 1095 | 421 | 674 | 0.935 |
| n (%) | 94 (8.6%) | 37 (8.7%) | 57 (8.5%) |  |
| FGA | 1081 | 411 | 670 | 0.510 |
| n (%) | 21 (1.9%) | 10 (2.4%) | 11 (1.6%) |  |
| Any antidepressant | 1380 | 526 | 854 | **<0.001** |
| n (%) | 611 (44.3%) | 194 (37.0%) | 417 (48.8%) |  |
| **Two or more antidepressants, *n*** | 1380 | 526 | 854 | **<0.001** |
| Yes, *n* (%) | 111 (8.0%) | 21 (4.0%) | 90 (10.5%) |  |
| SSRI | 1364 | 516 | 848 | **<0.001** |
| n (%) | 305 (22.4%) | 80 (15.5%) | 225 (26.5%) |  |
| SNRI | 1177 | 444 | 733 | 0.982 |
| n (%) | 123 (10.5%) | 45 (10.1%) | 78 (10.6%) |  |
| TCA | 1254 | 455 | 799 | 0.118 |
| n (%) | 45 (3.6%) | 12 (2.6%) | 33 (4.1%) |  |
| AD without MS | 1422 | 556 | 866 | **0.001** |
| n (%) | 181 (12.7%) | 43 (7.7%) | 138 (15.9%) |  |
| Thyroid hormone | 839 | 292 | 547 | 0.014 |
| n (%) | 174 (20.7%) | 81 (27.2%) | 93 (17.2%) |  |
| Stimulants/wakefulness | 1112 | 414 | 698 | 0.644 |
| n (%) | 117 (10.5%) | 41 (9.9%) | 76 (10.9%) |  |
| Dopamine agonist | 1130 | 414 | 716 | 0.091 |
| n (%) | 18 (1.6%) | 4 (1.0%) | 14 (2.0%) |  |
| Two or more SGA | 1405 | 548 | 857 | 0.889 |
| n (%) | 72 (5.1%) | 30 (5.5%) | 42 (4.9%) |  |
| One or more MS | 1420 | 556 | 864 | 0.092 |
| n (%) | 1003 (70.6%) | 426 (76.6%) | 577 (66.8%) |  |
| Two or more MSs | 1420 | 556 | 864 | 0.357 |
| n (%) | 158 (11.1%) | 64 (11.5%) | 94 (10.9%) |  |
| Three or more MSs | 1420 | 556 | 864 | 0.969 |
| n (%) | 1 (0.1%) | 0 (0.0%) | 1 (0.1%) |  |
| No MS | 1420 | 556 | 864 | 0.092 |
| n (%) | 417 (29.4%) | 130 (23.4%) | 287 (33.2%) |  |
| No medications | 1450 | 572 | 878 | 0.304 |
| n (%) | 112 (7.7%) | 34 (5.9%) | 78 (8.9%) |  |
| ***Lifetime prescriptions*** |  |  |  |  |
| Lithium ever | 1282 | 496 | 786 | 0.035 |
| n (%) | 736 (57.4%) | 307 (61.9%) | 429 (54.6%) |  |
| Lamotrigine ever | 1352 | 522 | 830 | 0.283 |
| n (%) | 538 (39.8%) | 192 (36.8%) | 346 (41.7%) |  |
| Valproic ever | 1357 | 523 | 834 | 0.053 |
| n (%) | 545 (40.2%) | 232 (44.4%) | 313 (37.5%) |  |
| Gabapentinoids ever | 1436 | 546 | 890 | 0.015 |
| n (%) | 187 (13.0%) | 51 (9.0%) | 136 (15.6%) |  |
| Benzodiazepines ever | 1327 | 505 | 822 | **<0.001** |
| n (%) | 723 (54.5%) | 236 (46.7%) | 487 (59.2%) |  |
| Non-BZD sedatives ever | 1316 | 502 | 814 | 0.138 |
| n (%) | 240 (18.2%) | 77 (15.3%) | 163 (20.0%) |  |
| SGA ever | 1405 | 548 | 857 | 0.992 |
| n (%) | 1032 (73.5%) | 394 (71.9%) | 638 (74.4%) |  |
| FGA ever | 1081 | 411 | 670 | 0.994 |
| n (%) | 126 (11.7%) | 52 (12.7%) | 74 (11.0%) |  |
| Any antidepressant ever | 1380 | 526 | 854 | 0.019 |
| n (%) | 1105 (80.1%) | 391 (74.5%) | 714 (83.5%) |  |
| SSRI ever | 1364 | 516 | 848 | **<0.001** |
| n (%) | 893 (65.5%) | 292 (56.6%) | 601 (70.9%) |  |
| SNRI ever | 1177 | 444 | 733 | 0.103 |
| n (%) | 391 (33.2%) | 125 (28.2%) | 266 (36.3%) |  |
| TCA ever | 1254 | 455 | 799 | 0.078 |
| n (%) | 239 (19.1%) | 70 (15.4%) | 169 (21.2%) |  |
| Thyroid hormone ever | 839 | 292 | 547 | 0.113 |
| n (%) | 75 (8.9%) | 35 (11.7%) | 40 (7.4%) |  |
| Stimulants/wakefulness ever | 1112 | 414 | 698 | 0.733 |
| n (%) | 275 (24.7%) | 89 (21.5%) | 186 (26.6%) |  |
| Dopamine agonist ever | 1130 | 414 | 716 | 0.561 |
| n (%) | 32 (2.8%) | 10 (2.4%) | 22 (3.1%) |  |
| AD without MS ever | 1419 | 555 | 864 | **<0.001** |
| n (%) | 165 (11.6%) | 40 (7.2%) | 125 (14.5%) |  |
| Two or more SGAs ever | 1405 | 548 | 857 | 0.707 |
| n (%) | 478 (34.0%) | 176 (32.1%) | 302 (35.2%) |  |
| One or more MS ever | 1420 | 556 | 864 | 0.060 |
| n (%) | 1190 (83.8%) | 483 (86.9%) | 707 (81.8%) |  |
| Two or more MSs ever | 1420 | 556 | 864 | 0.812 |
| n (%) | 522 (36.8%) | 207 (37.2%) | 315 (36.5%) |  |
| Three or more MSs ever | 1420 | 556 | 864 | 0.423 |
| n (%) | 185 (13.0%) | 76 (13.7%) | 109 (12.6%) |  |
| No MS ever | 1420 | 556 | 864 | 0.060 |
| n (%) | 230 (16.2%) | 73 (13.1%) | 157 (18.2%) |  |
| No medications ever | 1440 | 572 | 878 | 0.633 |
| n (%) | 21 (1.4%) | 8 (1.4%) | 13 (1.5%) |  |

*Models adjusted for age, sex, and recruitment site.

ANX: anxiety disorders; BZD: benzodiazepines; FGA: first-generation antipsychotics; MS: mood stabilizer (includes valproate, lamotrigine, carbamazepine, and lithium); SGA: second-generation antipsychotic; SNRI: serotonin-norepinephrine reuptake inhibitor; SSRI: selective serotonin reuptake inhibitor; TCA: tricyclic antidepressant.

**Supplementary Table 2**. Differences in pharmacotherapeutic prescriptions among individuals with bipolar disorder-II, with and without comorbid anxiety disorders (ANX).

| **Variable** | **Total**  **(N=723)** | **NoANX**  **(N=263)** | **ANX**  **(N=460)** | **p-value*** |
| --- | --- | --- | --- | --- |
| Lithium | 397 | 155 | 242 | 0.352 |
| n (%) | 153 (38.5%) | 64 (41.3%) | 89 (36.8%) |  |
| Lamotrigine | 524 | 191 | 333 | 0.313 |
| n (%) | 260 (49.6%) | 89 (46.6%) | 171 (51.4%) |  |
| Valproic | 477 | 177 | 300 | 0.452 |
| n (%) | 107 (22.4%) | 44 (24.9%) | 63 (21.0%) |  |
| Carbamazepine | 452 | 166 | 286 | 0.873 |
| n (%) | 11 (2.4%) | 4 (2.4%) | 7 (2.4%) |  |
| Gabapentinoids | 711 | 259 | 452 | 0.005 |
| n (%) | 52 (7.3%) | 9 (3.5%) | 43 (9.5%) |  |
| Benzodiazepines | 615 | 220 | 395 | **<0.001** |
| n (%) | 212 (34.5%) | 53 (24.1%) | 159 (40.3%) |  |
| Non-BZD sedatives | 615 | 220 | 395 | 0.579 |
| n (%) | 47 (7.6%) | 15 (6.8%) | 32 (8.1%) |  |
| Olanzapine | 418 | 156 | 262 | 0.342 |
| n (%) | 21 (5.0%) | 10 (6.4%) | 11 (4.2%) |  |
| Clozapine | 410 | 152 | 258 | 0.981 |
| n (%) | 1 (0.2%) | 0 (0.0%) | 1 (0.4%) |  |
| Quetiapine | 452 | 166 | 286 | 0.287 |
| n (%) | 132 (29.2%) | 42 (25.3%) | 90 (31.5%) |  |
| Aripiprazole | 428 | 158 | 270 | 0.159 |
| n (%) | 57 (13.3%) | 15 (9.5%) | 42 (15.6%) |  |
| Risperidone | 415 | 154 | 261 | 0.991 |
| n (%) | 26 (6.3%) | 9 (5.8%) | 17 (6.5%) |  |
| FGA | 460 | 171 | 289 | 0.981 |
| n (%) | 1 (0.2%) | 0 (0.0%) | 1 (0.3%) |  |
| SGA | 638 | 230 | 408 | 0.051 |
| n (%) | 262 (41.1%) | 81 (35.2%) | 181 (44.4%) |  |
| SSRI | 669 | 238 | 431 | <0.001 |
| n (%) | 182 (27.2%) | 45 (18.9%) | 137 (31.8%) |  |
| SNRI | 554 | 199 | 355 | 0.157 |
| n (%) | 100 (18.1%) | 30 (15.1%) | 70 (19.7%) |  |
| TCA | 597 | 212 | 385 | 0.853 |
| n (%) | 35 (5.9%) | 13 (6.1%) | 22 (5.7%) |  |
| Thyroid hormone | 318 | 121 | 197 | 0.324 |
| n (%) | 81 (25.5%) | 27 (22.3%) | 54 (27.4%) |  |
| Stimulants/wakefulness | 532 | 184 | 348 | 0.677 |
| n (%) | 79 (14.8%) | 25 (13.6%) | 54 (15.5%) |  |
| Dopamine agonist | 515 | 187 | 328 | 0.052 |
| n (%) | 6 (1.2%) | 5 (2.7%) | 1 (0.3%) |  |
| Any antidepressant | 677 | 239 | 438 | **<0.001** |
| n (%) | 379 (56.0%) | 105 (43.9%) | 274 (62.6%) |  |
| **Two or more antidepressants, *n*** | 677 | 239 | 438 | 0.118 |
| Yes, *n* (%) | 107 (15.8%) | 31 (13.0%) | 76 (17.4%) |  |
| AD without MS | 691 | 251 | 440 | 0.046 |
| n (%) | 117 (16.9%) | 33 (13.0%) | 84 (19.2%) |  |
| Two or more SGA | 638 | 230 | 408 | 0.069 |
| n (%) | 12 (1.9%) | 7 (3.0%) | 5 (1.2%) |  |
| One or more MS | 696 | 255 | 441 | 0.397 |
| n (%) | 472 (67.8%) | 179 (70.2%) | 293 (66.4%) |  |
| Two or more MSs | 696 | 255 | 441 | 0.860 |
| n (%) | 58 (8.3%) | 22 (8.6%) | 36 (8.2%) |  |
| Three or more MSs | 696 | 255 | 441 | 0.980 |
| n (%) | 1 (0.1%) | 0 (0.0%) | 1 (0.2%) |  |
| No MS | 696 | 255 | 441 | 0.397 |
| n (%) | 224 (32.2%) | 76 (29.8%) | 148 (33.6%) |  |
| No medications | 715 | 259 | 456 | 0.437 |
| n (%) | 69 (9.7%) | 27 (10.4%) | 42 (9.2%) |  |
| ***Lifetime prescriptions*** |  |  |  |  |
| Lithium ever | 551 | 204 | 347 | 0.325 |
| n (%) | 247 (44.8%) | 99 (48.5%) | 148 (42.7%) |  |
| Lamotrigine ever | 665 | 238 | 427 | 0.709 |
| n (%) | 327 (49.2%) | 113 (47.5%) | 214 (50.1%) |  |
| Valproic ever | 668 | 239 | 429 | 0.448 |
| n (%) | 201 (30.1%) | 77 (32.2%) | 124 (28.9%) |  |
| Carbamazepine ever | 666 | 239 | 427 | 0.887 |
| n (%) | 37 (5.6%) | 13 (5.4%) | 24 (5.6%) |  |
| Gabapentinoid ever | 711 | 259 | 452 | 0.015 |
| n (%) | 90 (12.7%) | 22 (8.5%) | 68 (15.0%) |  |
| Benzodiazepines ever | 615 | 220 | 395 | **<0.001** |
| n (%) | 309 (50.2%) | 83 (37.7%) | 226 (57.2%) |  |
| Non-BZD sedatives ever | 615 | 220 | 395 | 0.834 |
| n (%) | 121 (19.7%) | 43 (19.5%) | 78 (19.7%) |  |
| SGA ever | 638 | 230 | 408 | 0.003 |
| n (%) | 381 (59.7%) | 119 (51.7%) | 262 (64.2%) |  |
| FGA ever | 460 | 171 | 289 | 0.038 |
| n (%) | 18 (3.9%) | 3 (1.8%) | 15 (5.2%) |  |
| Any antidepressant ever | 677 | 239 | 438 | **<0.001** |
| n (%) | 567 (83.8%) | 183 (76.6%) | 384 (87.7%) |  |
| SSRI ever | 669 | 238 | 431 | 0.232 |
| n (%) | 449 (67.1%) | 150 (63.0%) | 299 (69.4%) |  |
| SNRI ever | 554 | 199 | 355 | 0.002 |
| n (%) | 241 (43.5%) | 70 (35.2%) | 171 (48.2%) |  |
| TCA ever | 597 | 212 | 385 | 0.105 |
| n (%) | 104 (17.4%) | 32 (15.1%) | 72 (18.7%) |  |
| AD without MS, ever | 697 | 247 | 450 | 0.279 |
| n (%) | 96 (13.8%) | 30 (11.8%) | 66 (14.9%) |  |
| Thyroid hormone ever | 318 | 121 | 197 | 0.154 |
| n (%) | 39 (12.3%) | 11 (9.1%) | 28 (14.2%) |  |
| Stimulants/wakefulness ever | 532 | 184 | 348 | 0.007 |
| n (%) | 150 (28.2%) | 37 (20.1%) | 113 (32.5%) |  |
| Dopamine agonist ever | 515 | 187 | 328 | 0.230 |
| n (%) | 11 (2.1%) | 6 (3.2%) | 5 (1.5%) |  |
| Two or more SGAs ever | 638 | 230 | 408 | 0.142 |
| n (%) | 136 (21.3%) | 40 (17.4%) | 96 (23.5%) |  |
| One or more MS ever | 696 | 255 | 441 | 0.518 |
| n (%) | 568 (81.6%) | 212 (83.1%) | 356 (80.7%) |  |
| Two or more MSs ever | 696 | 255 | 441 | 0.979 |
| n (%) | 182 (26.1%) | 66 (25.9%) | 116 (26.3%) |  |
| Three or more MSs ever | 696 | 255 | 441 | 0.512 |
| n (%) | 54 (7.8%) | 22 (8.6%) | 32 (7.3%) |  |
| No MS ever | 696 | 255 | 441 | 0.518 |
| n (%) | 128 (18.4%) | 43 (16.9%) | 85 (19.3%) |  |
| No medications ever | 715 | 259 | 456 | 0.328 |
| n (%) | 15 (2.1%) | 7 (2.7%) | 8 (1.8%) |  |

*Models adjusted for age, sex, and recruitment site.

ANX: anxiety disorders; BZD: benzodiazepines; FGA: first-generation antipsychotics; MS: mood stabilizer (includes valproate, lamotrigine, carbamazepine, and lithium); SGA: second-generation antipsychotic; SNRI: serotonin-norepinephrine reuptake inhibitor; SSRI: selective serotonin reuptake inhibitor; TCA: tricyclic antidepressant.
